# Glucocorticoids in the Management of Bronchial Asthma: From Pathophysiology to Precision Medicine

**DOI:** 10.3390/biomedicines14102181

**Published:** 2026-09-26

**Authors:** Andrada Anamaria Stâncoi, Annamaria Pallag, Cristina Burlou-Nagy (Fati), Diana Maria Burța, Beata Lelesz, Diana Bei, Tünde Horvath

**Affiliations:** 1 Doctoral School of Biological and Biomedical Sciences, University of Oradea, Universitatii Str. No 1, 410087 Oradea, Romania; stancoi.andradaanamaria@student.uoradea.ro (A.A.S.); burlounagy.cristina@student.uoradea.ro (C.B.-N.); burta.dianamaria@student.uoradea.ro (D.M.B.); 2 Department of Pharmacy, Faculty of Medicine and Pharmacy, University of Oradea, P-ta 1 Decembrie No 10, 410073 Oradea, Romania; thorvath@uoradea.ro; 3 Department of Pharmacology and Pharmacotherapy, Faculty of Medicine, University of Debrecen, Nagyerdei Krt 98, 4032 Debrecen, Hungary; lelesz.beata@med.unideb.hu; 4 Department of Medical Disciplines, Faculty of Medicine and Pharmacy, University of Oradea, 410028 Oradea, Romania; diana.bei@didactic.uoradea.ro

**Keywords:** bronchial asthma, airway inflammation, glucocorticoids, inhaled corticosteroids, asthma endotypes, corticosteroid resistance, precision medicine, biologic therapies

## Abstract

Bronchial asthma is a heterogeneous chronic inflammatory airway disease characterized by diverse pathogenic mechanisms and variable responses to therapy. This review article analyzes the essential role of glucocorticoids in asthma management, starting with the pathophysiological mechanisms involved in the progression of the disease and the characteristics of the Th2-high and Th2-low endotypes. The molecular and cellular mechanisms by which glucocorticoids regulate airway inflammation, reduce bronchial hyperresponsiveness, and improve clinical outcomes are discussed. Furthermore, the main forms of glucocorticoid administration—namely, inhaled, oral, and injectable formulations—are described, along with their integration into current recommended treatment strategies for the various severity levels of asthma. Particular attention is given to the phenomenon of glucocorticoid resistance, the factors involved in its development, and its impact on treatment efficacy. The article provides an analysis of the safety profile of glucocorticoid therapy, including adverse reactions, limitations on use, and the main contraindications. In conclusion, current trends and future prospects for optimizing the treatment of bronchial asthma are presented, with an emphasis on personalized glucocorticoid therapies and the identification of new therapeutic targets capable of improving disease control and patient outcomes.

## 1. Introduction

Bronchial asthma is a chronic inflammatory condition of the airways involving numerous cells and cellular components, particularly: mast cells, eosinophils, T lymphocytes, T cells, macrophages, neutrophils, and epithelial cells [1]. In susceptible individuals, this inflammation can cause frequent episodes of wheezing, shortness of breath, a burning sensation in the chest, and coughing, especially at night or in the morning. These episodes are often associated with widespread but variable airflow limitation, with obstruction that is often either reversible or resolves spontaneously with treatment [1,2].

Inflammation can also exacerbate existing bronchial hyperreactivity in response to a variety of stimuli. The reversibility of airflow limitation may be insufficient in some patients with asthma [1].

The cause of bronchial asthma is unknown, but numerous risk factors for the disease have been identified, with gene-environment interactions being particularly important. Genetics is known to influence asthma, with heritability ranging from 35% to 95%. Genetic studies have shown that there are hundreds of genetic variants associated with an increased risk of asthma [3].

Respiratory infections, especially viral infections early in life, can lead to an increased risk of developing asthma, particularly when symptoms are severe. Exposure to a certain unfavorable environment increases the risk of bronchial asthma, including factors such as tobacco smoke or air pollutants [1,4].

Atypical conditions and sensitization, as well as numerous inhaled allergens, are also associated with the development of asthma. Other factors that promote the development of asthma have also been identified, such as the effects of the microbiome, vitamin D, chemical exposures, certain changes in diet, stress, and metabolites [5,6,7].

Understanding a complex condition such as asthma requires accounting for a high level of genetic diversity, which is expressed variably due to environmental influences, epigenetic and transcriptional factors, leading to certain histopathological characteristics that are less variable and resulting in cardinal asthmatic symptoms [1].

Bronchial asthma is a chronic inflammatory disease of the airways, characterized by significant clinical and immunological heterogeneity, which leads to substantial differences in therapeutic response. Currently, glucocorticoids remain the primary class of medications used to control bronchial inflammation and reduce exacerbations; however, their efficacy may vary depending on the molecular mechanisms involved and the disease phenotype [6,8].

The aim of this paper is to analyze the role of glucocorticoids in the management of bronchial asthma by integrating recent data from the scientific literature regarding the pathogenic mechanisms of the disease, the characteristics of Th2-high and Th2-low inflammation, as well as the mechanisms of action of glucocorticoids at the cellular level. At the same time, the paper evaluates the main forms of glucocorticoids used in clinical practice, current therapeutic strategies, the mechanisms involved in the development of treatment resistance, and the limitations associated with corticosteroid therapy. Finally, it presents modern therapeutic approaches and future prospects for optimizing the treatment of bronchial asthma [8].

The literature identification and study selection process is summarized in Figure 1 to improve the transparency and reproducibility of the structured literature search conducted for this review. The structured search identified 1573 records, of which 167 studies fulfilled the predefined eligibility criteria and were included in the qualitative synthesis presented in this review [8].

## 2. Pathological Mechanisms in Bronchial Asthma

Classifying disease endotypes based on fundamental pathophysiological mechanisms has become a logical approach in asthma, as an endotype-based strategy allows for better diagnostic evaluation, monitoring, and stratification of patients [9,10]. In severe asthma, endotypes are differentiated based on the primary immuno-inflammatory pathway involved, with high type 2 (T2), low type 2 (non-T2), and mixed forms, which share multiple common pathophysiological mechanisms, including genetic and epigenetic, metabolic, neurogenic, and tissue remodeling subtypes [11,12,13,14].

### 2.1. Mechanisms of Th2-High Asthma

Th2 cells constitute a distinct subpopulation of effector CD4+ T helper cells, characterized by the secretion of the cytokines IL-4, IL-5, IL-13, and IL-9. Th2-dependent inflammation is responsible for approximately half of all cases of mild-to-moderate asthma, as well as for a significant proportion of severe forms of the disease [15,16]. Since the mechanisms of Th2-high asthma have been extensively described in the literature, this section highlights the main pathophysiological processes involved [16,17,18].

The Th2-type inflammatory response unfolds in two essential stages. The first stage, sensitization, occurs when inhaled allergens reach the lower airways and are processed by antigen-presenting cells, which subsequently activate Th2 cells. These cells release specific cytokines, particularly IL-4, IL-5, and IL-13. IL-4 and IL-13 induce the activation of B lymphocytes and the production of immunoglobulin E (IgE), which binds to FcεRI receptors on the surface of mast cells. The second stage, known as the allergen challenge, occurs upon re-exposure to the same antigen. Binding of the allergen to IgE triggers mast cell degranulation and the release of pro-inflammatory mediators, including histamine, leukotrienes, and cytokines. Concurrently, allergens can stimulate cholinergic nerves, promoting the release of acetylcholine. These chemical mediators and neurotransmitters cause bronchial smooth muscle contraction and the onset of bronchoconstriction [15,16,17].

IL-5 plays a central role in the differentiation, maturation, and recruitment of eosinophils in the lungs [19]. Once activated, eosinophils release major basic protein (MBP), which amplifies inflammation by stimulating mast cells and increasing the release of histamine and leukotrienes. In addition, MBP inhibits M2 muscarinic receptors, which promotes the release of acetylcholine from cholinergic nerve endings and exacerbates bronchospasm [20]. At the same time, IL-13 directly contributes to the hypersensitization of bronchial smooth muscle, stimulates mucin secretion by the respiratory epithelium, and promotes airway remodeling and fibrosis [21].

The respiratory epithelium plays an active role in initiating and maintaining Th2-type inflammation by secreting cytokines in response to exposure to harmful agents such as infections, pollutants, or tissue injury. These cytokines include TSLP, IL-25, and IL-33, which activate type 2 innate lymphoid cells (ILC2). ILC2 produce significant amounts of IL-5 and IL-13, thereby contributing to the amplification of Th2 pulmonary inflammation [15,16]. There is also evidence that IL-33 exerts direct effects on mast cell activation, bronchial smooth muscle migration, and the expression of the clinical phenotype of asthma, as seen in Figure 2 [22].

Th9 cells and the cytokine IL-9 represent another important component of Th2 inflammation. Although Th9 cells can secrete several cytokines, IL-9 is considered the primary mediator involved in the pathogenesis of asthma. Due to its pleiotropic nature, IL-9 influences multiple cell types, including T and B lymphocytes, mast cells, and macrophages. By activating Th2 cells and increasing mast cell accumulation, IL-9 contributes to the intensification of Th2-type inflammation [23].

In addition, IL-9 can activate Arg1+ interstitial macrophages, which release the chemokine CCL5, promoting the recruitment of eosinophils, T cells, and monocytes into the lung parenchyma and perpetuating type 2 inflammation [24].

Natural killer T (NKT) cells are a lymphocyte subpopulation present in large numbers in the lungs and lymphoid tissues. Some studies suggest that these cells may contribute to Th2 inflammation by secreting IL-4 and IL-13 or by facilitating the production of these cytokines by Th2 cells [25]. However, the data available in the literature are contradictory, and other studies do not support a major role for NKT cells in the pathogenesis of asthma [26,27].

### 2.2. Mechanisms of Th2-Low Asthma

The definition of Th2-low asthma is not based on the identification of specific phenotypic features, but rather on the absence of inflammatory and immunological markers characteristic of Th2-high asthma. These markers include elevated fractional exhaled nitric oxide (FeNO), increased eosinophil counts in blood and sputum, and high serum levels of total immunoglobulin E. This approach is widely accepted and used in clinical practice, primarily because it is based on noninvasive, readily available, and reproducible biomarkers [28,29].

Data from studies conducted in real-world clinical settings indicate that patients with T2-low asthma tend to be older, with a late onset of the disease, frequently associated with obesity, and exhibit poor symptom control, an increased incidence of exacerbations, and a reduced therapeutic response to corticosteroids [30].

Interleukin-17 is considered an important mediator in the pathogenesis of Th2-low asthma [31,32,33]. Evidence suggests that genetic variations in components involved in the IL-17 pathway may influence susceptibility and disease progression. Clinical studies have demonstrated significant increases in IL-17 concentrations in serum, sputum, and bronchoalveolar lavage fluid in patients with asthma, with these levels positively correlated with clinical severity [34,35].

IL-17 is produced by a variety of cell types, of which T helper 17 (Th17) cells are a major source. At the same time, IL-17 secretion has also been identified in other cell populations, including CD8+ T lymphocytes, natural killer T cells, mucosa-associated invariant T (MAIT) cells, type 3 innate lymphoid cells, and B lymphocytes, highlighting the complexity of the immune network involved, as seen in Figure 3 [36].

Although the exact role of IL-17 cytokines in asthma is not fully understood, existing data indicate that they can activate epithelial cells and fibroblasts, inducing the secretion of chemokines with chemotactic effects on neutrophils, such as CXCL1, CXCL5, and CXCL8, as well as granulocyte-macrophage colony-stimulating factor [37]. These processes promote neutrophil infiltration into the airways, a mechanism characteristic of non-T2 inflammation. In addition, IL-17A, unlike IL-17F, exerts direct effects on bronchial smooth muscle, promoting its contraction, migration, and proliferation, thereby contributing to the development of bronchial hyperreactivity and airway remodeling [38].

However, some studies suggest that IL-17 may also play a beneficial role by helping to maintain the integrity of the respiratory epithelium and potentially exerting protective effects in certain contexts of asthma [36].

Th1 cells produce cytokines such as IL-2, interferon-γ (IFN-γ), and lymphotoxin-α, which contribute to cell-mediated immune responses. Although traditionally associated with Th1-driven immunity, these cytokines may also participate in asthma pathogenesis. TNF-α, a pleiotropic pro-inflammatory cytokine, is increased in the sputum of patients with neutrophilic and severe asthma and may act synergistically with IL-17 to promote neutrophil recruitment. TNF-α may additionally enhance the production of IL-4, IL-5, and IL-13 and increase airway smooth muscle contractility, thereby contributing to airway hyperresponsiveness (AHR) [36].

IFN-γ, IL-1β, and TNF-α can also increase CD38 (cluster of differentiation 38) expression in airway smooth muscle cells, enhancing intracellular Ca^2++^ signaling and promoting AHR. Consistently, CD38 deficiency has been associated with reduced AHR in murine asthma models [30]. IFN-γ may further contribute to neutrophilic inflammation by promoting neutrophil recruitment in the presence of IL-17 [36].

## 3. The Role of Glucocorticoids in Bronchial Asthma

### 3.1. Glucocorticoids: Mechanism of Action

Inhaled corticosteroids are the most effective agents for controlling asthma. They primarily inhibit inflammation by suppressing several activated inflammatory genes through the reversal of histone acetylation via the recruitment of histone deacetylase-2 [39].

Inhaled corticosteroids reduce airway hyperresponsiveness and control asthma symptoms by suppressing airway inflammation. Inhaled corticosteroids are now the first-line treatment for all patients with persistent bronchial asthma, to control asthma symptoms and prevent exacerbations [39].

Long-acting inhaled β2-agonists (LABAs), when used in combination with inhaled corticosteroids (ICS), provide complementary bronchodilator effects and can further improve asthma control [39].

In contrast, inhaled corticosteroids have significantly lower clinical efficacy in COPD, and inflammation is resistant to the effects of corticosteroids. This may be due to decreased activity and expression of histone diacetylase-2 as a result of oxidative stress [40].

Inhaled corticosteroids are used in combination with bronchodilators in patients with severe COPD to reduce the number of exacerbations (Table 1) [39]. Inhaled corticosteroids, which are absorbed from the lungs into the systemic circulation, have negligible systemic side effects at the doses required for most patients; however, at higher doses in COPD, they are associated with systemic side effects and an increased risk of pneumonia [40].

Major advances have been made in understanding the molecular mechanisms by which inhaled corticosteroids suppress inflammation in asthma, based on recent developments in the fundamental understanding of gene transcription mechanisms. Corticosteroids activate and suppress many genes that are relevant to understanding their efficacy in asthma. There has also been progress in understanding the molecular mechanisms of resistance to inhaled corticosteroids in severe asthma and COPD [40,41].

### 3.2. Effects at the Cellular Level

In patients with asthma who are sensitive to glucocorticoids (GCs), these medications exert a marked anti-inflammatory effect on the airways, acting on both immune cells and structural cells of the respiratory tree [42,43]. A relevant example is the reduction in eosinophil survival through the inhibition of granulocyte-macrophage colony-stimulating factor (GM-CSF) expression [42]. Furthermore, corticosteroids (GCs) suppress lymphocyte activation and induce their apoptosis in vitro, while simultaneously reducing the number of circulating lymphocytes in patients with asthma receiving oral treatment [43].

GC treatment also affects other immune cell populations. Thus, macrophage activity is diminished by the inhibition of cytokine and chemokine secretion, while monocytes show a reduction in number in the peripheral blood, as well as low expression of IgE receptors [43]. Furthermore, GCs modulate the migration of dendritic cells to regional lymph nodes. By regulating the expression of adhesion molecules and the production of chemokines, GC limits the recruitment and infiltration of immune cells into peripheral tissues [44,45].

In addition to their effects on the immune system, GCs significantly influence the function of bronchial smooth muscle (ASM) cells, a key element in the pathogenesis of asthma [46]. Previous studies have demonstrated that GCs can suppress the expression of several proinflammatory genes involved in airway remodeling and hyperreactivity [47]. In this regard, dexamethasone and fluticasone propionate have been reported to inhibit TNF-induced production of relevant chemokines and cytokines, including CXCL8 [48,49], CCL5, IL-6 [50,51], CCL11 [52], and CXCL10 [53], as well as the expression of the adhesion molecule ICAM-1 [54].

Furthermore, inflammatory responses triggered by IL-1β—such as the activation or expression of matrix metalloproteinase 12 (MMP-12), the production of CXCL10 and GM-CSF, or the induction of ICAM-1—are also attenuated by treatment with dexamethasone or fluticasone [54,55,56,57,58]. GCs have also been shown to be effective in inhibiting the release of proinflammatory mediators, such as IL-6 and CXCL8, generated following the activation of G-protein-coupled receptors by agonists such as bradykinin or sphingosine-1-phosphate [59,60,61,62].

Similarly, ciclesonide and fluticasone propionate show comparable efficacy in suppressing TNF-induced CCL2 expression [63,64]. However, the inhibitory effects of GCs on proinflammatory genes in ASM cells are not uniform. Certain genes, such as IL-6, CCL2, CCL5, and CXCL10, are almost completely suppressed at high pharmacological concentrations, while others, including ICAM-1, CXCL8, CCL11, and GM-CSF, are only partially inhibited [65,66,67,68].

### 3.3. The Therapeutic Role and Comparative Efficacy of Inhaled and Systemic Glucocorticoids in Asthma

Inhaled and systemic glucocorticoids have different therapeutic roles in asthma, determined mainly by disease severity and the clinical setting. Inhaled glucocorticoids are primarily used to control airway inflammation, while systemic glucocorticoids are generally reserved for severe exacerbations and selected patients with severe asthma requiring additional anti-inflammatory treatment [69,70].

In the current paradigm, where asthmatic inflammation is believed to cause pathological tissue remodeling, anti-inflammatory treatment with glucocorticoids should reduce the rapid and progressive loss of lung function in people with asthma [71,72].

Glucocorticoids are hormones synthesized in the adrenal cortex and secreted into the bloodstream, and glucocorticoid levels fluctuate in a circadian cycle. In humans, the natural glucocorticoid is hydrocortisone (cortisol), which is synthesized from its precursor, cortisone [70]. The beneficial effects of glucocorticoids on patients with bronchial asthma were first reported in 1950. Since then, many studies have focused on the therapeutic potential of glucocorticoids. Several glucocorticoids have been synthesized that are much more effective than cortisol and do not have the undesirable side effects of mineralocorticoids [73].

### 3.4. Inhaled Glucocorticoids

Inhaled corticosteroids (ICS) are indicated for the prevention of asthma exacerbations in patients with persistent bronchial asthma. Persistent asthma is characterized by symptoms occurring more than two days per week, waking up at night more than three times per month, using short-acting beta-2 adrenergic agonists twice a week or more frequently to control symptoms, or any restriction of normal activity due to asthma [74,75]. Regular use of these medications improves quality of life by reducing the frequency of bronchial asthma symptoms, bronchial hyperreactivity, and the increased risk of severe exacerbations [76].

These medications are introduced gradually, depending on the frequency and severity of asthma symptoms. Inhaled corticosteroids can be administered in low, medium, and high doses for the treatment of mild, moderate, and severe persistent asthma, as seen in Table 2 [77,78]. When asthma symptoms remain insufficiently controlled with ICS therapy, additional controller treatment may be considered, such as the addition of a long-acting β2-agonist (LABA) or, in selected patients, a leukotriene receptor antagonist (LTRA) [79].

These medications are delivered directly to the site of action via inhalation. This method of administration avoids first-pass metabolism, which occurs with oral administration, thereby reducing the dose required to achieve the desired effect [80].

Side effects are also minimized due to reduced systemic bioavailability. Inhaled corticosteroids are available as liquid formulations, capsules administered via a nebulizer, metered-dose inhalers used with a spacer, and dry powder inhalers [81,82].

The timing of inhaled corticosteroid administration may influence therapeutic efficacy in bronchial asthma, supporting the concept of chronotherapy. Pincus et al. demonstrated that once-daily administration of inhaled triamcinolone at 3:00 p.m. resulted in improvements in lung function comparable to those achieved with conventional four-times-daily administration, without additional systemic corticosteroid effects [83]. More recently, Wang et al. investigated the influence of ICS administration timing in a randomized, crossover study with three treatment regimens and found that administration of beclomethasone dipropionate in the mid-afternoon, 400 μg between 3:00 p.m. and 4:00 p.m., was associated with a more pronounced improvement in nocturnal lung function and inflammatory biomarkers compared with once-daily administration in the morning and twice-daily administration [84]. These results highlight the potential importance of considering the timing of ICS administration in optimizing corticosteroid therapy for bronchial asthma [84].

### 3.5. Oral Glucocorticoids

Oral corticosteroids (OCS) have been the standard of care for the treatment of acute asthma exacerbations for several decades, with their use having been established as early as the second half of the 20th century [85]. Evidence from systematic reviews confirms their important role in controlling these episodes by reducing the risk of relapse, decreasing the need for short-acting beta-2 agonists (SABA) [86], and significantly lowering the rate of hospitalizations during the acute phase [87].

Beyond their use in acute exacerbations, OCS may also be required in a subset of patients with severe uncontrolled asthma who continue to experience symptoms despite optimized treatment with high-dose ICS and LABA therapy [88,89,90].

However, chronic use of OCS is associated with an increased risk of severe side effects and contributes significantly to rising healthcare costs [91,92]. Furthermore, recent data indicate that even repeated, short-term use for acute exacerbations can lead to a cumulative effect of side effects [92,93,94]. This issue is particularly relevant, given that a significant percentage of patients with severe asthma require at least two courses of OCS annually to control exacerbations [95,96,97].

Prior to the introduction of modern therapies, more than half of patients with uncontrolled severe asthma required maintenance treatment with OCS in addition to standard therapy. Currently, multidimensional assessment and the introduction of innovative adjunctive therapies can reduce the frequency of exacerbations and, consequently, dependence on OCS [95]. Furthermore, certain biologic therapies have demonstrated the ability to reduce the need for OCS in severe asthma. However, the still-high use of these medications suggests that the implementation of these therapeutic strategies is not yet optimal in clinical practice [98,99,100].

### 3.6. Injectable Glucocorticoids

Injectable glucocorticoids are used in emergency situations, specifically during asthma attacks, as well as in patients who do not respond favorably to treatment with beta-2 agonists inhalers. They are almost exclusively administered intravenously. They work by suppressing the immune system, thereby reducing inflammation in the body, as seen in Table 3 [101].

Injectable glucocorticoids are used because they provide a rapid therapeutic effect, generally faster than that of oral forms. They are administered exclusively in a hospital setting to treat severe exacerbations. Intravenous administration results in much faster absorption and an immediate effect, reducing inflammation in the airways [101,103].

After treatment of a severe asthma attack, the administration of injectable glucocorticoids can help prevent possible relapses and maintain symptom control, in the short term. In some cases, patients with severe bronchial asthma require hospitalization, for continuous monitoring and intensive treatment [104].

## 4. Current Treatment Strategies for Bronchial Asthma

The management of bronchial asthma requires an individualized approach aimed at reducing inflammation, controlling symptoms, and preventing exacerbations. In clinical practice, treatment is structured in stages, with the intensity adjusted according to the severity of the disease and the patient’s response [105].

Short-acting β2-agonists (SABAs) act rapidly on bronchial smooth muscle to induce bronchodilation and relieve acute asthma symptoms. Although they improve airflow by reducing bronchoconstriction, they do not treat the underlying airway inflammation and therefore have a limited role in controlling the inflammatory component of asthma [106,107]. To achieve effective long-term control, SABA are often replaced by or combined with long-acting β2-agonists (LABA), administered in combination with corticosteroids [108]. LABA provide prolonged bronchodilation, helping to alleviate nocturnal symptoms and improve lung function. However, their use as monotherapy is contraindicated, as they do not affect underlying inflammation and may lead to its exacerbation [109]. In some cases, adjunctive therapies, such as vitamin D supplementation, may also be used to support the immune response [110,111].

Inhaled corticosteroids (ICS) are the cornerstone of background therapy in asthma, due to their pronounced anti-inflammatory effect. They act through glucocorticoid receptors, inhibiting the production of proinflammatory cytokines, reducing the recruitment of inflammatory cells, and contributing to the restoration of bronchial epithelial integrity [112]. Their efficacy is particularly evident in mild and moderate forms of the disease, where they improve symptom control and lung function. In contrast, in severe asthma or in cases associated with glucocorticoid resistance, the introduction of additional therapies is often necessary. Systemic corticosteroids, administered orally or intravenously, are mainly reserved for severe asthma exacerbations and selected patients with severe disease requiring additional anti-inflammatory treatment. Their long-term use is limited by the risk of systemic adverse effects [113].

Leukotriene receptor antagonists (LTRA) represent a therapeutic alternative, acting on leukotriene-mediated inflammation [114]. They are particularly useful in certain asthma phenotypes, such as exercise-induced asthma or asthma associated with aspirin intolerance, reducing bronchoconstriction, mucus secretion, and inflammation by blocking specific receptors in the airways. In recent years, biologic therapies have revolutionized the treatment of severe forms of asthma, particularly those characterized by specific inflammatory mechanisms. Biologic therapies have expanded the treatment options available for patients with severe asthma, particularly those with defined inflammatory phenotypes. Monoclonal antibodies such as mepolizumab and benralizumab target the IL-5 pathway and reduce eosinophilic inflammation [115]. Similarly, dupilumab inhibits IL-4 and IL-13 signaling and targets type 2 inflammation [116].

These therapies are particularly valuable in patients with glucocorticoid-resistant asthma, offering an effective alternative and allowing for a reduction in the need for systemic corticosteroids [117]. Their development reflects the transition toward personalized medicine, in which treatment is tailored to the individual characteristics of the disease. Currently, ongoing advances in the field of biologic therapies and combination therapies are contributing to improved outcomes for patients with severe or refractory asthma [118].

Asthma comprises distinct inflammatory patterns that can influence both corticosteroid responsiveness and therapeutic choices. Patients with T2-high asthma commonly present with elevated eosinophils, FeNO, or IgE and may show a better response to corticosteroid treatment. In T2-low asthma, the absence of established T2 biomarkers is often accompanied by a reduced response to corticosteroids. In severe disease, biomarker assessment can further support the selection of targeted biologic therapies, as seen in Table 4 [119].

## 5. Glucocorticoid Resistance

This syndrome is a rare genetic disorder that can occur both in families and sporadically, and it is characterized by generalized, partial insensitivity of target tissues to the action of glucocorticoids [120]. As a result of reduced glucocorticoid sensitivity in the central components of the hypothalamic–pituitary–adrenal (HPA) axis, the negative feedback mechanism acting on the hypothalamus and anterior pituitary is diminished [121].

This dysfunction leads to a compensatory increase in hypothalamic secretion of corticotropin-releasing hormone (CRH) and arginine vasopressin (AVP), as well as stimulation of ACTH secretion in the anterior pituitary. Excess ACTH leads to bilateral adrenocortical hyperplasia and increased production and secretion of cortisol, as a compensatory mechanism for its reduced efficacy at the tissue level [121].

At the same time, elevated ACTH levels also stimulate the synthesis of other adrenal steroids, including mineralocorticoids (such as deoxycorticosterone and corticosterone) and adrenal androgens (androstenedione, dehydroepiandrosterone—DHEA—and its sulfate—DHEA-S). Since tissue sensitivity to these hormones remains intact, their excess leads to specific clinical manifestations [121].

Therefore, elevated levels of mineralocorticoids can lead to the development of hypertension and/or hypokalemic alkalosis, while excess adrenal androgens can cause clinical manifestations either through direct effects on target tissues or indirectly, by modulating the hypothalamic-pituitary-gonadal axis (Figure 4) [121,122].

Excess adrenal androgens in patients with this syndrome are associated with multiple clinical manifestations, such as acne, hirsutism (predominantly in women), reduced fertility in both sexes, androgenic alopecia, menstrual cycle disorders, and oligo-anovulation in women, and oligospermia in men [123]. During childhood, accelerated bone maturation may be observed, which subsequently correlates with reduced final height. In female newborns, genital differentiation abnormalities may occur, including clitoromegaly or ambiguous genitalia [124].

Clinical signs suggestive of glucocorticoid deficiency are rare in adults, but they can occur in newborns and young children, where they manifest as severe episodes of hypoglycemia, sometimes complicated by seizures or coma, given the essential role of glucocorticoids in supporting hepatic gluconeogenesis during the first years of life [125,126,127]. In some adult patients, symptoms such as anxiety or chronic fatigue may occur, likely caused by increased hypothalamic secretion of CRH and/or AVP [128,129,130].

Elevated circulating ACTH concentrations are associated with the development of bilateral adrenal hyperplasia [131,132]. Additionally, incidental adrenal masses may be identified in some patients [133]. Although these lesions are common in the general population, prolonged exposure to elevated levels of ACTH may contribute to their initiation or progression. In addition, a case of an ACTH-secreting pituitary adenoma has been reported, possibly facilitated by excessive stimulation via CRH and/or AVP [134].

The clinical spectrum of the syndrome is heterogeneous, ranging from severe forms to mild or even asymptomatic forms, in which only biochemical changes are present [131]. This variability is primarily determined by the differing impact of mutations on glucocorticoid receptor function, but may also be influenced by additional factors, such as individual genetic characteristics, epigenetic mechanisms, and biochemical changes associated with age and lifestyle [135,136].

### 5.1. Corticosteroid Resistance in Severe Asthma

Leukocyte redistribution following systemic corticosteroid administration may represent a potential biomarker of treatment response and corticosteroid resistance in severe asthma. The study, conducted on 142 patients, identified two distinct groups: patients with more pronounced leukocyte redistribution showed a better response to corticosteroids, while patients with reduced redistribution exhibited characteristics associated with lower sensitivity to corticosteroids, such as an increased frequency of B cells and more intense IgE sensitization to multiple allergens. These results suggest that dynamic changes in leukocyte populations following corticosteroid administration may help identify patients with resistance to corticosteroid treatment [137].

### 5.2. Reduced Corticosteroid Responsiveness Seen in Severe Asthma

The use of oral corticosteroids (OCS) remains common in severe asthma, despite therapeutic advances, and is associated with significant and long-lasting systemic adverse effects. Biologic therapies with monoclonal antibodies can reduce both the frequency of OCS-treated exacerbations and the need for corticosteroids in OCS-dependent patients. Management of these patients involves identifying corticosteroid-related comorbidities, optimizing and gradually reducing the OCS dose after initiating biologic therapy, and monitoring hypothalamic–pituitary–adrenal axis function to prevent adrenal insufficiency [138].

The response to glucocorticoids is heterogeneous in severe asthma and is largely influenced by the inflammatory endotype of the disease. Patients with type 2 (T2) asthma, characterized by eosinophilia and the involvement of the cytokines IL-4, IL-5, and IL-13, generally exhibit better responsiveness to glucocorticoids. In contrast, non-T2 phenotypes, frequently associated with neutrophilic inflammation, obesity, or smoking, may exhibit a reduced response to corticosteroids and greater resistance to treatment. At the molecular level, glucocorticoid resistance may be associated with overexpression of the GRβ isoform of the glucocorticoid receptor, impaired nuclear translocation of GRα via activation of the MAPK pathway, and oxidative stress-induced inactivation of histone deacetylase 2 (HDAC2). These mechanisms may diminish the anti-inflammatory action of glucocorticoids and contribute to persistent inflammation and inadequate disease control, necessitating intensified treatment and, in certain cases, the use of biologic therapies that conserve corticosteroids [139].

Inhaled glucocorticoids form the basis of asthma treatment due to their anti-inflammatory effects. They act by binding to glucocorticoid receptors (GRs), which subsequently translocate into the nucleus and regulate the expression of genes involved in the inflammatory response. Abnormal activation of certain signaling pathways and transcription factors, such as NF-KB, can impair glucocorticoid receptor function and reduce the response to corticosteroids. These changes contribute to the development of glucocorticoid resistance, which is particularly common in severe forms of asthma [140].

## 6. Adverse Effects, Limitations, and Contraindications in the Use of Glucocorticoids

Glucocorticoids constitute a first-line therapeutic class used in the management of a wide range of conditions, including allergic, inflammatory, rheumatological, oncological, vascular, and dermatological diseases, as well as other systemic pathologies [141,142,143]. However, prolonged use, particularly at high doses, is frequently associated with the onset of severe adverse effects, predominantly of a metabolic and atrophic nature. These include musculoskeletal system impairment, manifested as glucocorticoid-induced osteoporosis, osteonecrosis, and myopathy, as well as disturbances in carbohydrate and lipid metabolism, including diabetes mellitus, dyslipidemia, and obesity [144,145]. Adverse effects on the gastrointestinal and cardiovascular systems have also been reported [146].

Despite significant advances in research, there is currently no clear consensus on the selection of optimal animal models that faithfully reproduce the pathogenic mechanisms underlying glucocorticoid-induced adverse effects [147]. The identification and validation of relevant and reproducible animal models are essential for further understanding the mechanisms involved in glucocorticoid toxicity, as well as for the development and evaluation of new glucocorticoid receptor ligands, particularly selective glucocorticoid receptor agonists (SEGRAs). Both glucocorticoids (GCs) and selective glucocorticoid receptor agonists (SEGRAs) exert their effects through the glucocorticoid receptor (GR), a transcription factor belonging to the nuclear receptor superfamily. GR activation following binding to glucocorticoids or their analogs involves two main mechanisms of transcriptional regulation. On the one hand, DNA-independent transrepression (TR) is primarily responsible for the therapeutic effects of GR ligands by inhibiting the expression of proinflammatory genes [148].

On the other hand, DNA-dependent transactivation (TA) involves the formation of GR dimers that bind to glucocorticoid response elements, leading to the activation of genes involved in the development of adverse reactions. This mechanism is associated with the development of complications such as diabetes mellitus, peptic ulcer, Cushing’s syndrome, osteoporosis, hyperglycemia, as well as skin and muscle atrophy, along with other undesirable systemic effects [148].

Acute psychosis may occur in patients taking high-dose glucocorticoids. The first step is to immediately discontinue the medication upon the onset of symptoms. Although various medications have been tried—including antipsychotics, antidepressants, benzodiazepines, and hydrocortisone—with varying degrees of success, there is still a lack of consensus regarding the optimal therapy to halt and reverse the neuropsychiatric side effects caused by corticosteroids in adults or children. The adverse reactions caused by these medications further contribute to a reduction in their use [149,150].

Contraindications for corticosteroid administration include: allergic reactions to any ingredient in the formulation, concurrent use of live or attenuated vaccines (when immunosuppressive doses are used), systemic fungal infections, osteoporosis, uncontrolled hyperglycemia, diabetes mellitus, glaucoma, joint infections, uncontrolled hypertension, herpes simplex keratitis, and varicella infection. Other relative contraindications include peptic ulcer, congestive heart failure, and viral or bacterial infections that are not being managed with anti-infective treatments, as seen in Table 5 [151,152,153].

Children and adolescents require special attention due to the risk of growth suppression. An assessment of pubertal stage and nutritional status is necessary for any child or adolescent who is to be treated with corticosteroids [152].

## 7. Current and Future Treatment Perspectives

For over four decades, inhaled glucocorticoids (ICS) have been an essential component of asthma management, with their therapeutic role firmly supported by scientific evidence [154]. Their impact extends beyond reducing mortality, significantly altering the global course of the disease, such that hospitalizations for asthma have become increasingly rare [155].

However, the safety profile of ICS remains an important consideration. Adverse effects, such as adrenal insufficiency, metabolic disorders, skin fragility, oral candidiasis, decreased bone density, or impaired growth in children, are well documented. Even though their incidence is low, they can influence perceptions of the treatment. Despite this, the benefit-risk ratio remains favorable for the long-term use of these medications [155].

A relevant issue is low treatment adherence, often driven by concerns about adverse reactions, both among patients and, at times, among physicians. In this context, the development of new formulations of inhaled glucocorticoids, characterized by an optimized pharmacological profile, could reduce these limitations [156,157]. Such an approach requires a detailed understanding of the mechanisms of action, particularly the non-genomic ones and the effects of genomic transactivation, which are thought to be involved in the occurrence of adverse reactions. In this regard, modified analogs with improved therapeutic potential have recently been proposed [158].

Furthermore, prolonging the half-life of these drugs could help improve treatment adherence. Previous experience with intramuscular administration of extended-release triamcinolone has demonstrated benefits for adherence; however, the use of this strategy is limited by its unfavorable adverse reaction profile [159].

The management of bronchial asthma is based primarily on the use of inhaled glucocorticoids (ICS), which have an anti-inflammatory effect, and beta-2 agonists, which are responsible for the bronchodilator effect [160,161]. In recent years, treatment strategies have evolved significantly, with current guidelines recommending the combined use of these two classes of medications even in mild forms of the disease, starting at age 6. This change was driven by evidence showing that the exclusive use of short-acting beta-2 agonists is associated with an increased risk of severe exacerbations and mortality [162,163].

Therapies that include inhaled glucocorticoids, whether administered continuously or as needed, are considered the most effective in preventing asthma exacerbations across all age groups [164,165,166]. For adolescents and adults, the ICS-formoterol combination is preferred for both maintenance and rescue therapy due to its ability to significantly reduce the frequency of exacerbations [167].

For children aged 6 to 11 years with persistent asthma, treatment options are more varied, including both ICS monotherapy and combinations of ICS with long-acting beta-2 agonists (LABAs) or fixed-dose ICS-formoterol combinations, with specific characteristics depending on the treatment step [167,168,169].

The clinical studies used in developing the guidelines evaluated the efficacy of various combinations, such as fluticasone propionate compared with the fluticasone/salmeterol combination, and budesonide compared with the budesonide/formoterol combination, highlighting the benefits of combination therapy [170,171].

## 8. Precision Medicine and New Treatment Strategies for Bronchial Asthma

Bronchial asthma is a chronic inflammatory disease of the airways, associated with bronchial hyperreactivity and affecting hundreds of millions of people worldwide. Advances in recent decades have significantly improved disease control, and an understanding of asthma phenotypes and endotypes has facilitated the transition to precision medicine. The development of biologic therapies targeting mediators such as IgE, TSLP, IL-4, IL-5, and IL-13 has helped reduce exacerbations and the need for oral corticosteroids, as well as improve lung function and quality of life. The identification of new therapeutic targets continues to support the development of increasingly personalized strategies for the management of severe asthma [172].

Recent advances in drug-delivery technologies have explored the possibility of achieving more targeted pulmonary delivery of glucocorticoids in asthma. Although inhaled corticosteroids already provide preferential delivery to the respiratory tract, conventional formulations may be limited by incomplete deposition at sites of inflammation, mucociliary clearance, and potential systemic exposure. Consequently, nanoparticle-based systems and other advanced carriers have been investigated to improve pulmonary retention and facilitate drug delivery to specific airway cells [173].

In experimental models of asthma, dexamethasone-loaded nanoparticles designed to interact with the airway epithelium have demonstrated enhanced pulmonary retention and anti-inflammatory activity compared with conventional formulations [174]. Similarly, an inhalable dendrimer–dexamethasone conjugate targeting inflammatory cells reduced eosinophilic infiltration and inflammatory responses in a model of allergic asthma [175]. These findings suggest that molecular or cellular targeting could potentially improve the therapeutic index of glucocorticoids by increasing drug exposure at sites of airway inflammation while limiting systemic distribution. However, current evidence remains predominantly preclinical, and important challenges concerning pulmonary deposition, formulation stability, pharmacokinetics, safety, and translation to patients remain [172,173]. Therefore, targeted glucocorticoid delivery represents a promising but still investigational strategy in asthma [172,173,174].

### Delivery Systems Based on Lipogels and Nanogels in Bronchial Asthma

Liposomal formulations represent a promising approach for drug delivery in asthma, offering advantages over conventional formulations, such as increased biocompatibility, prolonged pulmonary retention of the active ingredient, reduced extrapulmonary adverse effects, and improved therapeutic efficacy [176,177].

In this context, Honmane et al. developed a liposomal dry-powder inhaler for salbutamol sulfate, which provided sustained drug release for up to 14 h and exhibited improved aerosol properties compared to conventional formulations. The results suggest that this strategy may help prolong the therapeutic effect and reduce the frequency of administration [178].

A similar approach was investigated by Chen et al., who developed a liposomal aerosol formulation of salbutamol sulfate. Compared to the free form, the liposomal formulation exhibited prolonged release and maintained the antiasthmatic effect for a longer period in the experimental model, highlighting the potential of liposomes to increase pulmonary retention and the duration of drug action [179].

Elhissi et al. also demonstrated the feasibility of developing liposomal formulations that combine salbutamol sulfate with beclomethasone dipropionate, suggesting the possibility of using them for the simultaneous pulmonary administration of a bronchodilator and a corticosteroid [180].

The liposomal delivery of corticosteroids is also of particular interest. Konduri et al. demonstrated, in an experimental model of allergic asthma, that weekly administration of budesonide encapsulated in “stealth” liposomes reduced markers of pulmonary inflammation, including eosinophilia, serum IgE levels, and the cytokines IL-4 and IL-5, with efficacy comparable to that of daily budesonide administration [181]. These results suggest that liposomal encapsulation may prolong the action of corticosteroids and reduce the frequency of administration [182].

In addition, Yin Ng et al. investigated curcumin-loaded liposomes, observing a reduction in pro-inflammatory markers such as IL-6, IL-8, IL-1β, and TNF-α in a cellular model. Although these results support the therapeutic potential of liposomal curcumin in asthma, further studies are needed to confirm the efficacy and clinical relevance of this approach [182].

Inhalation administration is an important therapeutic strategy in bronchial asthma, as it allows for the direct delivery of active substances to the airways, with the potential to increase local bioavailability, accelerate the onset of the therapeutic effect, and limit systemic exposure and first-pass hepatic metabolism [183,184]. In this context, the development of nanotechnological drug delivery systems has attracted considerable interest. Nanogels can improve the solubility and bioavailability of poorly soluble active substances and can facilitate their cellular penetration and absorption [184,185]. Their application in pulmonary delivery can also offer advantages such as controlled and sustained drug release, reduced frequency of dosing, and optimization of therapeutic efficacy and patient compliance [186,187,188].

Among the materials used to produce nanogels, alginate is of particular interest due to its natural origin, biodegradability, biocompatibility, and properties that make it suitable for the formulation of drug delivery systems [189,190,191]. It can form gel-like structures through cross-linking with divalent metal ions, with calcium being frequently used due to its stability and safety profile [192]. Furthermore, the use of zinc ions for alginate cross-linking is of additional interest due to their attributed anti-inflammatory properties [191,192]. However, the development of zinc alginate-based nanogels remains relatively unexplored, partly due to the need for specific preparation conditions and adequate technical expertise.

## 9. Conclusions

Bronchial asthma is a complex and heterogeneous condition in which the diversity of pathogenic mechanisms and inflammatory patterns leads to significant variations in disease progression and response to treatment. Glucocorticoids remain the cornerstone of anti-inflammatory therapy due to their broad-spectrum effects at the molecular and cellular levels; however, their efficacy is not uniform, particularly in certain forms of asthma and in the context of treatment resistance.

Although their benefits are well documented, the use of glucocorticoids is limited by potential adverse effects and the need for careful adjustment of therapy. In this context, a deeper understanding of the mechanisms involved in the pathogenesis of asthma has led to the development of modern therapeutic options, including targeted therapies, which can improve disease control in patients with severe or difficult-to-treat forms of the disease.

Glucocorticoids can be used as targeted therapies for bronchial asthma, particularly through the development of modern pharmaceutical formulations that allow for more effective and selective delivery to the lungs. These strategies can improve inflammation control while reducing systemic exposure and the risk of adverse effects. Integrating these technologies into precision medicine can help personalize treatment for patients with asthma.

The current management of bronchial asthma is moving toward an individualized approach aimed at optimizing therapeutic efficacy and reducing treatment-related risks, with the goal of improving patients’ quality of life and long-term disease control.

## Figures and Tables

**Figure 1:**
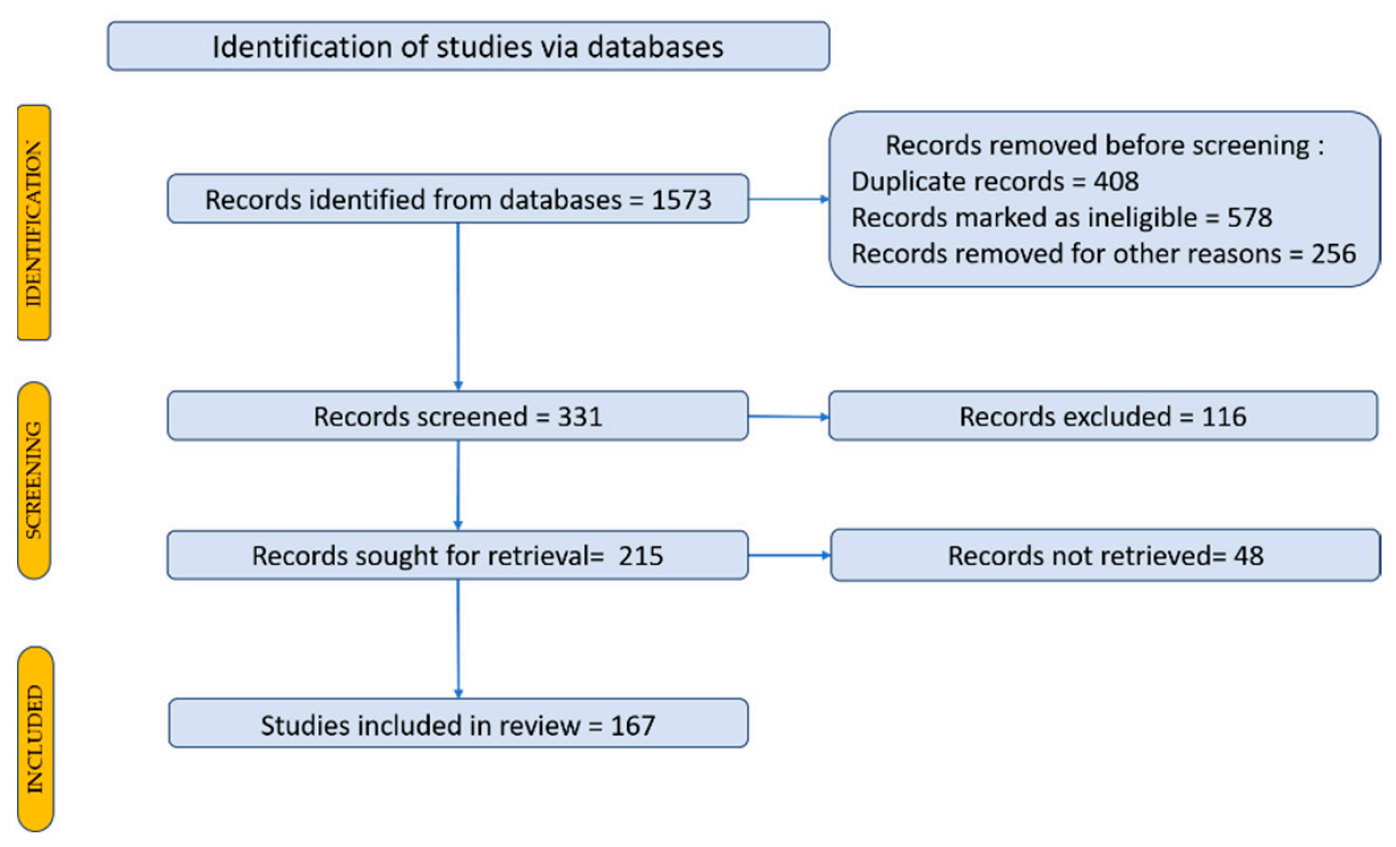
Flow diagram illustrating the literature search and study selection process for the present review [8].

**Figure 2:**
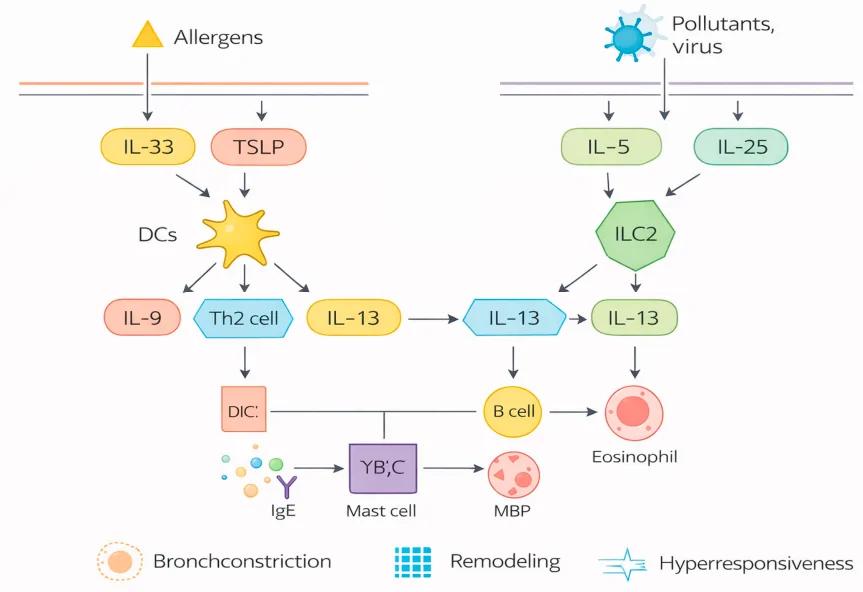
Conceptual diagram of airway inflammation mediated by the type 2 immune response. Exposure to allergens and environmental factors triggers the activation of the respiratory epithelium and the release of epithelial alarmins (IL-33, TSLP, IL-25), which initiate innate and adaptive immune responses. Subsequent signaling via type 2 cytokines (IL-4, IL-5, IL-13) leads to IgE production, eosinophilic inflammation, and the activation of effector cells, promoting bronchoconstriction, airway remodeling, and bronchial hyperreactivity [22].

**Figure 3:**
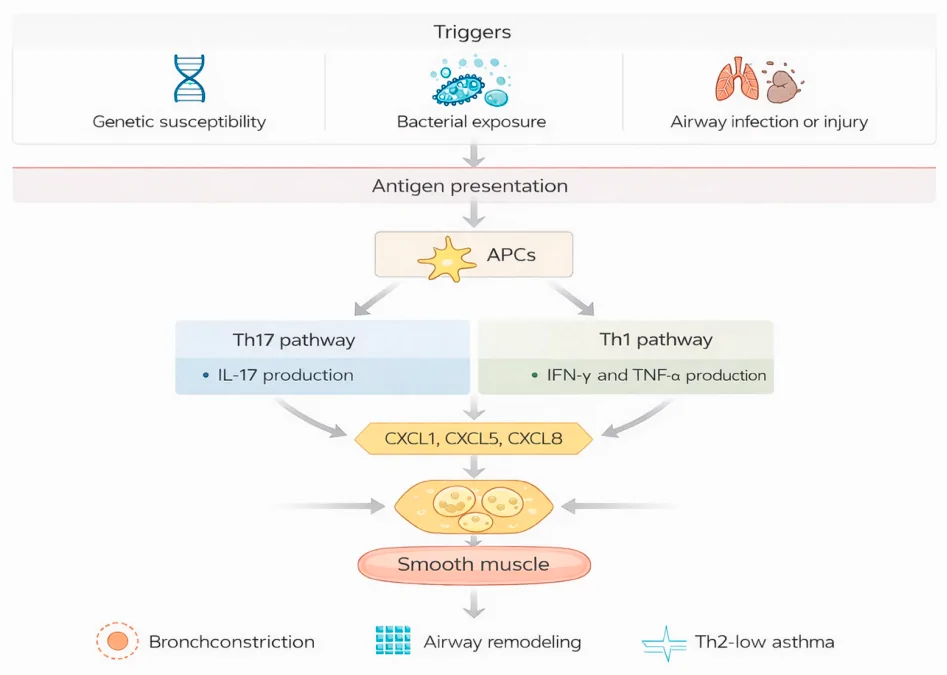
The mechanism of Th2-low asthma involves the activation of Th17 and Th1 pathways. Exposure to bacteria stimulates the differentiation of Th17 cells via antigen-presenting cells, and IL-17 triggers the release of chemokines (CXCL1/5/8), promoting neutrophil recruitment and contributing to airway remodeling and hyperreactivity. At the same time, infection and epithelial damage induce a Th1 response, with secretion of TNF-α and IFN-γ, which amplify inflammation, increase bronchial smooth muscle contractility, and intensify neutrophil recruitment, particularly in synergy with IL-17 [36].

**Figure 4:**
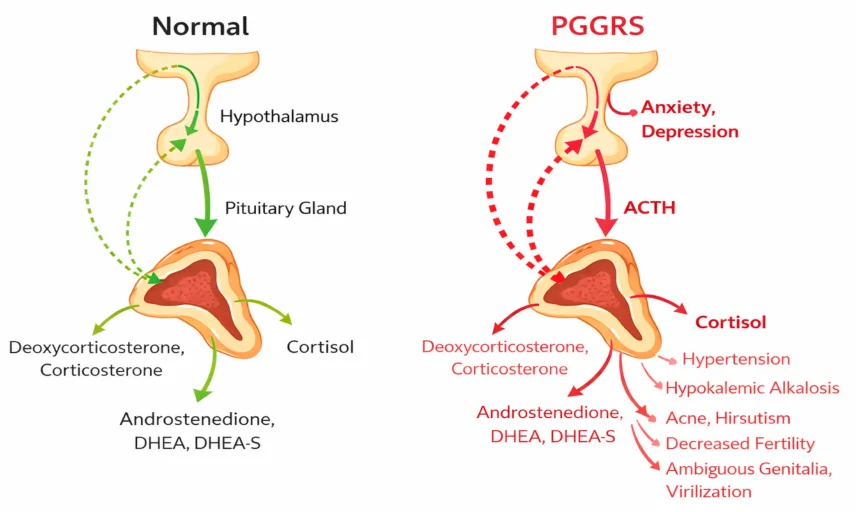
The pathophysiological mechanisms of PGGRS involve generalized glucocorticoid resistance, which leads to compensatory hyperactivation of the HPA axis, with increased secretion of CRH/AVP, ACTH, and cortisol. Excess ACTH further stimulates the synthesis of mineralocorticoids and adrenal androgens, which are responsible for the clinical manifestations, while the effects of excess cortisol are limited due to tissue insensitivity [122,123].

**Table 1:** The effect of glucocorticoids on gene transcription [39].

Increased Transcription	Decreased Transcription
Lipocortin-1	Inflammatory cytokines-IL-2, IL-3, IL-4, IL-5, IL-6, IL-11, IL-13, IL-15, TNFa, GM-CSF, SCF
B2-adrenergic receptors	Chemokines- IL-8, RANTES, MIP-1a, eotaxin
Leukocyte-secreting inhibitory protein	Inflammatory enzymes- Inducible nitric oxide synthase (iNOS), inducible cyclooxygenase (COX-2), inducible phospholipase A2 (cPLA2)
IxB-a (NF-kB inhibitor)	Inflammatory peptides- Endothelin-1
Anti-inflammatory or inhibitory cytokines: IL-10, IL-12, IL-1 receptor antagonist	Mediator receptors- neurokinin (NKI) and bradykinin (B2) receptors
Mitogen-activated protein kinase phosphatase-1 (MKP-1, inhibits MAPK pathways)	Adhesion molecules- ICAM-1, VCAM-1

**Table 2:** This table presents both the differences between the three types of inhaled dosage forms and their advantages and disadvantages [77].

	Nebulizer	Metered-Dose Inhalers	Dry Powder Inhalers
Advantages	No need to coordinate breathing with the timing of medication administration Ability to administer higher doses Ideal for children, the elderly, and people with breathing Provide long-term and sustained treatment, making them beneficial during severe exacerbations	Are portable and easy to carry Are not as expensive as nebulizers Quick administration Precise and consistent delivery of the medication dose	Require no coordination between inhalation and triggering the inhaler, since the device is activated by inhalation They do not contain propellants, thus reducing the risk of throat irritation The device is simple and easy to use
Disadvantages	The devices are more bulky and less portable compared to other inhalers Administration time is longer Requires regular maintenance for proper hygiene	Requires coordination between inhalation and the moment the inhaler is triggered Some propellants may cause throat irritation or a sensation of cold It is difficult to administer a higher dose	Requires a much deeper inhalation to obtain an adequate dose of medication In conditions of high humidity, they may be less effective because the powder clumps together Cannot be used in mechanically ventilated patients

**Table 3:** Oral corticosteroids are effective in controlling acute asthma exacerbations, but their long-term use is limited by the risk of adverse reactions. Currently, modern therapies help reduce the need for them [102].

Acute Exacerbation	Long-Term Use	Limitation and Risks	Current Approach
Reduce the risks of symptom recurrence Reduce the need for rescue medication (SABA) Decrease the frequency of hospitalizations	Indicated for severe forms of uncontrolled asthma Used when inhaled therapy does not provide adequate control	May cause significant adverse reactions Cumulative effect, including after repeated courses of treatment Associated with increased medical costs	Biological therapies offer modern alternatives Help reduce the need for OCS

**Table 4:** Relationship between asthma inflammatory patterns, biomarkers, corticosteroid responsiveness, and therapeutic implications [119].

Asthma Inflammatory Pattern	Main Biomarkers/ Features	Expected Corticosteroid Response	Therapeutic Implications
T2-high asthma	Elevated eosinophils, FeNO and/or IgE	Generally better corticosteroid responsiveness	Corticosteroid treatment; targeted biologic therapies in severe disease
T2-low asthma	Absence of established T2 biomarkers	Reduced corticosteroid responsiveness	Alternative/targeted therapeutic approaches may be considered
Severe asthma	Biomarker assessment may help characterize inflammatory profile	Response may vary according to inflammatory pattern	Biomarker-guided selection of targeted biologic therapies

**Table 5:** The contraindications for glucocorticoids, ↑ indicates an increase in activity, while ↓ indicates a decrease in activity [153].

Contraindications	Mechanism
Peptic ulcer	↑ Irritation of the gastric mucosa, ↓ protection (bicarbonate and mucus)
Diabetes	↑ Insulin resistance and ↓ glucose utilization in muscles
Hypertension	↑ Water and salt retention and blood pressure
Pregnancy	↑ Risk of gestational diabetes and hypertensive complications
Infections (TB, fungal infections, viral infections)	Decreased immunity → increased risk of infections
Osteoporosis	↓ Calcium absorption and bone demineralization
Mental disorders/epilepsy disturbances	Lower seizure threshold and mood disturbances.
Heart/kidney failure	↑ Water and salt retention, metabolic imbalances

## Data Availability

No new data were created or analyzed in this study. Data availability is therefore not applicable.
